# Tailoring inputs to achieve maximal neuronal firing

**DOI:** 10.1186/2190-8567-1-3

**Published:** 2011-05-03

**Authors:** Jiaoyan Wang, Willie Costello, Jonathan E Rubin

**Affiliations:** 1 Department of Mathematics, Tianjin University of Technology and Education, Tianjin, 300222, People’s Republic of China; 2 Department of Philosophy, University of Toronto, Toronto, Ontario, Canada; 3 Department of Mathematics, University of Pittsburgh, Pittsburgh, PA 15260, USA

## Abstract

We consider the constrained optimization of excitatory synaptic input patterns to maximize spike generation in leaky integrate-and-fire (LIF) and theta model neurons. In the case of discrete input kicks with a fixed total magnitude, optimal input timings and strengths are identified for each model using phase plane arguments. In both cases, optimal features relate to finding an input level at which the drop in input between successive spikes is minimized. A bounded minimizing level always exists in the theta model and may or may not exist in the LIF model, depending on parameter tuning. We also provide analytical formulas to estimate the number of spikes resulting from a given input train. In a second case of continuous inputs of fixed total magnitude, we analyze the tuning of an input shape parameter to maximize the number of spikes occurring in a fixed time interval. Results are obtained using numerical solution of a variational boundary value problem that we derive, as well as analysis, for the theta model and using a combination of simulation and analysis for the LIF model. In particular, consistent with the discrete case, the number of spikes in the theta model rises and then falls again as the input becomes more tightly peaked. Under a similar variation in the LIF case, we numerically show that the number of spikes increases monotonically up to some bound and we analytically constrain the times at which spikes can occur and estimate the bound on the number of spikes fired.

## Introduction

A major component of theoretical neuroscience is the study of how various neuronal models respond to synaptic inputs. Indeed, chemical synaptic transmission offers a specific mechanism for the encoding of information that an organism senses from the external environment, filtered by the internal state of the organism. The functions performed by particular neurons and neuronal networks are in part determined by the nature of the inputs that they receive and are in part a result of the responses they generate to these inputs, due to their intrinsic properties. Thus, understanding neuronal input-output transformations represents a centrally important scientific goal.

Although the framework for incorporating synaptic inputs into computational models is well established, and the computational implications of such inputs have received significant attention, optimization problems involving synaptic inputs are not well represented in the literature. In this paper, we consider such a problem, namely what is the optimal way to tailor synaptic inputs, subject to certain constraints, to maximize the number of spikes that a neuron will fire?

In fact, we consider two variations on this problem, one based on maximizing the total number of spikes fired and one focused on maximal firing within a prescribed time interval. There are several reasons that maximizing numbers of spikes may be a biologically relevant neuronal goal. Since neurons operate under conditions in which efficient resource use could be evolutionarily advantageous, it could be useful if, subject to some constraint on the amount of input that is available, the synaptic input time course could be tailored to achieve the largest possible number of spikes. Certainly, there are brain areas, including areas of visual cortex and somatosensory cortex, where it appears that intensity of firing encodes stimulus information, with neurons showing maximal firing under optimally preferred stimulus conditions [1-3]. Similarly, a sufficiently high firing rate within a given time window may be needed to overcome inhibition or to outcompete activity of other neurons to influence a downstream readout neuron (e.g., [4-6] and many more recent works). Which input time courses yield maximal spiking will depend on the intrinsic properties of a neuron or neuronal model, and characterizing optimal input time courses for models may provide information about which neuronal coding functions they are well suited to represent.

In an earlier paper, a calculus of variations approach was used to determine the input current of minimal amplitude that causes a neuron to spike at a specified target time [7]. That work considered a phase model for a spiking neuron, with the evolution of phase *x *∈ [0, 2*π*) given by

where the impact of the input current *I*(*t*) is modulated by the phase sensitivity function *Z*(*x*). Similarly, earlier work analyzed optimal weak inputs to start or stop repetitive spiking in a general biological oscillator, with dynamics expressed in polar coordinates as(1)

with *ε *small [8]. Particular biological systems with dynamics qualitatively equivalent to those of (1) were also considered as examples to illustrate a calculus of variations approach to this problem, involving numerical solution of a system of ordinary differential equations obtained through the introduction of Lagrange multipliers. To our knowledge, ours is the first work to focus on the optimization of inputs, subject to particular constraints, to maximize the number or rate of neuronal output spikes fired.

To initiate research in this direction, we consider input optimization in two well-known mathematical models for single neurons, the leaky integrate-and-fire (LIF) model and the theta model [9-11], the mathematical formulations of which are given in Section 2. Both of these are scalar models, which allows us to use certain analytical approaches, rather than relying entirely on numerics, and we tune both models to be silent in the absence of inputs. One approach that we follow is to treat synaptic inputs as events with discrete onset times, which yields a phase plane representation of the co-evolution of an intrinsic neuronal variable and a synaptic input variable, as introduced previously for the LIF and theta models [12]. This approach would apply equally well in parameter regimes for which the models are intrinsically oscillatory, rather than silent, but we stick with the richer silent case. The LIF model was also considered by Börgers and Kopell, who proved (1) if a collection of phase-shifted, identical time-periodic inputs cause a spike in an LIF neuron, then the same inputs will also cause a spike if they are synchronized, and (2) if a constant input of size *A *causes a spike, then any time-periodic input with time average *A *will also cause a spike [13]. Both of these results show that the power of inputs to induce spikes in the LIF model increases as the input time course is made less uniform. Our results represent an extension of this idea, providing information about specific time courses that are optimal, not just for the generation of a single spike but for maximizing spike outputs. While the LIF model is a reasonable representation for a passive neuron, the theta model can be rigorously derived as a normal form for Type I spiking neurons [9-11], which feature a transition from silence to oscillations through a SNIC bifurcation [14]. Thus, consideration of the theta model allows us to explore how our results extend to a case with additional biological relevance. Interestingly, although both models can fire spikes at arbitrarily low frequencies, differences in their responses to inputs have been noted in previous work [12], which further motivates us to continue to compare them here.

In addition to discrete inputs, we also consider a continuous input formulated so that a particular parameter shapes the input time course and ask how that parameter should be tuned to achieve the maximal number of spikes within a given time window. The absence of a threshold and reset in the theta model is convenient for the application of variational techniques in this case. For the LIF model, we do not apply such techniques, but we nonetheless derive some results about the influence of the input shape parameter on the spike train that results from the input. We shall see that certain properties of each model observed in the discrete case carry over to the continuous case, yet there are some differences worth noting as well.

The remainder of the paper is organized as follows. In Section 2, we analyze the LIF model with discrete synaptic kicks, the cumulative sizes of which are constrained. We introduce phase plane structures that are useful for analysis of the resulting optimization problem and discuss some example strategies for controlling the timing of inputs before moving on to prove some results about these structures and optimal strategies. In particular, we show that two different scenarios are possible, depending on model parameters, and these yield different optimal input strategies. We also show how analytical estimates for the number of spikes fired can be derived for any given input time course. In Section 3, a somewhat similar analysis strategy is applied to the theta model with discrete synaptic kicks. We find that the phase plane structures for the theta model resemble one of the possible cases for the LIF model, with a corresponding similarity in optimal strategies. In Section 4, we turn to the analysis of continuous input for the theta model, seeking a maximal number of spikes in a fixed time window. We define the continuous input so that its shape can be modulated by a parameter yet its integral over all positive time is independent of the value of that parameter. Variational techniques yield a boundary value problem that we solve numerically to find locally and globally optimal parameter values. In Section 5, we consider the continuous input optimization problem for the LIF model, for which the discontinuous reset prevents application of the same variational techniques. We provide direct analysis showing that all spikes must be fired within a particular time interval and characterize the behavior of this interval as the input parameter is varied. Moreover, we prove that the number of spikes saturates as this parameter increases. Some similar analysis for the theta model, showing that spiking is lost as this parameter increases, appears in the Appendix. We conclude with a discussion in Section 6, where we summarize our results and discuss the novelty and relevance of our findings in the neuroscience setting.

## Integrate-and-Fire (LIF) Model with Synaptic Kicks

### Model

We consider the dynamics of an LIF model neuron with excitatory synaptic inputs as governed by the equations(2)(3)

together with the reset condition(4)

Equation (2) can be derived from a conductance-based equation

 with fixed intrinsic current conductances *g_j_*, but we think of it as a nondimensionalized abstract model in which voltage intrinsically converges to a baseline *I *and *E *is the reversal potential of a synaptic input with strength *g *> 0. We assume *v_r _*<*I *<*v_th_*, such that no spikes are fired in the absence of input, and *E *>*v_th_*, and we consider the invariant half-plane {*g *≥ 0} within the (*v*, *g*) phase plane, where (*I*, 0) is the unique stable critical point. Further, we represent the excitatory input by the equations(5)(6)

for *k_n _*∈ (0, *G*], *n *= 1, 2,... with *N *≥ 1 finite and *G *> 0 fixed in **R**. Eqs. (3) and (5) show that each input kick can be chosen to arrive at any time and instantaneously updates the value of *g *when it arrives and that the synaptic conductance *g *always decays exponentially between kicks. Eq. (6) states that the sum of all inputs, however they may be divided, is always equal to a fixed input allowance *G*. In the subsequent subsections, we assume that *I*, *E*, *v_th_*, *v_r_*, and *β *are fixed and consider how to partition and time the input *G *to yield the greatest number of threshold crossings, or spikes. Without loss of generality, we take *v_r _*= 0. First, we discuss a phase plane representation of this problem and consider some example strategies.

### Phase Plane Structures and Basic Strategies

**Figure 1:**
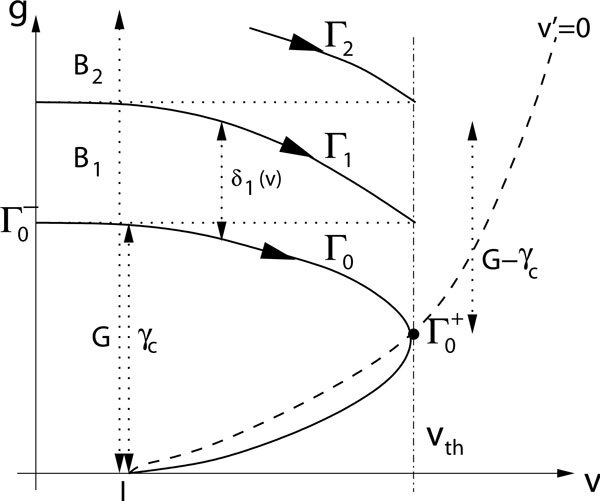
**Key structures in the phase plane for system (2),(3)**. Note that *G*, *v_th_*, and *I *are given parameters, while the other structures depend on the system dynamics.

We illustrate some key structures in the phase plane for system (2),(3) in Figure 1. The *v*-nullcline, based on equation (2), is the curve {g = (*v *- *I*)/(*E *- *v*)}. Denote the trajectory through the point  on this curve by Γ_0_, which is tangent to the line {*v *= *v_th_*}. Γ_0 _partitions the set {(*v*, *g*): *v *<*v_th_*, *g *≥ 0} into a set of initial conditions that yield spikes, namely those above Γ_0_, and a set of initial conditions that do not yield spikes, namely those below Γ_0_. Let  denote the intersection of Γ_0 _with {*v *= *v_r_*} and let  denote its *g *coordinate. The point  is on the threshold line, and there is a trajectory Γ_1 _that flows through this point. The band between Γ_0 _and Γ_1_, with *v_r _*≤ *v *≤ *v_th_*, consists of the set of initial conditions from which trajectories yield exactly one spike. Denote this band by *B*_1_. Similarly, for each natural number *n*, we can define a curve Γ*_n_*, such that each trajectory with an initial condition in the band *B_n _*between Γ_*n *-1 _and Γ*_n _*yields *n *spikes. Note that this band structure exists if parameters are altered such that *I *>*v_th_*, although the minimal band is shifted to negative *g *values, and hence the methods that we discuss easily generalize to this case.

The band structure that characterizes sets of initial conditions that yield particular numbers of spikes calls to mind several natural strategies for doling out input kicks to maximize spike output:

#### Critical kicks

Once a trajectory is below Γ_0_, it approaches (*I*, 0) asymptotically. Let *γ_c _*denote the *g*-coordinate of the intersection {*v *= *I*} ∩ Γ_0_. One possible strategy is to give an initial input of size *γ_c _*as well as subsequent inputs of size γ_c _each time the trajectory reaches a sufficiently small neighborhood of (*I*,0); see Figure 1. This *critical kicks *strategy yields *n *spikes where *nγ_c _*≤ *G *< (*n *+ 1)*γ_c_*.

#### Big kick

A possible disadvantage of the critical kicks strategy is that the increment *γ_c _*to achieve a spike may exceed the width *γ_n _*between bands Γ*_n _*and Γ_*n*-1 _for *n *≥ 1. To ensure that this increment is only encountered once, taking inspiration from the power of synchronized inputs [12,13], another reasonable strategy is to give a single *big kick *of size *G*, all at once, to the resting cell (Figure 1).

#### Reset and kick

An input of size *γ_c _*is sufficient to push the voltage across the threshold for single spike initiation. In the big kick strategy, the additional available input *G *- *γ_c _*is provided together with the *γ_c_*. It is possible that this additional input could deliver more spikes if it were delivered separately from the initial *γ_c_*. We can define a strategy, for example, in which an initial kick of size *γ_c _*is given to elicit a spike. As soon as this spike is fired, the cell is reset and the remaining input allowance *G *- *γ_c _*is given. Clearly, this *reset and kick *strategy would make sense if the bands *B_n _*were narrowest at *v *= 0.

#### Threshold kick

At the other extreme, the bands *B_n _*might be narrowest at *v *= *v_th_*. In this case, a possible optimal strategy would be the *threshold kick *strategy, defined by giving the initial kick of size *γ_c _*and following this with a kick of size *G *- *γ_c _*just before threshold crossing occurs (Figure 1).

Intuitively, it is reasonable to think that if *β *is large, such that inputs rapidly decay, then it makes sense to dole out inputs in minimal pieces, such that something like the critical kicks strategy may be optimal. Alternatively, if *β *is small, such that inputs decay slowly, then it makes sense to make inputs available as early as possible, such that one of other strategies is likely to be optimal. To analyze more carefully which strategy is optimal, it will be helpful to define a *band width δ_n_*(*v*) as the distance from Γ_*n*-1 _to Γ*_n _*in the *g*-direction for each fixed *v *∈ [0, *v_th_*]. With this definition in hand, we note that *δ*_*n*+1_(*v_th_*) = *δ*_*n*_(*v_r_*) for n ≥ 1, and hence the reset and kick and threshold kick strategies are effectively the same strategy, yielding the same number of spikes (Figure 1). We also let *δ*_∞_(*v*) = lim_*n*→∞ _*δ*_*n*_(*v*), *v *∈ [0, *v_th_*], if this limit exists. Very roughly speaking, the critical kicks strategy will yield approximately *G*/*γ_c _*spikes while the other strategies will induce about *G*/*δ*_∞_(*v*) spikes for some *v*, at least if *δ*_*n*_(*v*) converges to *δ*_∞_(*v*) quickly. Thus, comparison of *γ*_*c *_and *δ*_∞ _can be used to give an initial suggestion of what strategy to follow.

The value of *γ_c _*can be observed numerically by backwards integration from  until *v *= *I*. Alternatively, it may be optimal to replace *γ_c _*by the distance , as can be computed by backwards integration from  up to , and give kicks of size  after each spike, after the initial kick of size *γ_c_*; we will still refer to this as a critical kicks strategy.

An approximate value of *δ*_∞_(*v*) can be derived as follows. From (2),(3), the slope s of the vector field at any point in the phase plane is given by(7)

For *v *∈[*v_r_,v_th_*],

The magnitude of the change in *g *over one spike cycle is(8)

By construction, *δ*_∞_(*v_r_*) = *δ*_∞ _as given by (8). But since the value of *g *at reset for the trajectory forming the upper bound of one band is the value of *g *at threshold for the trajectory forming its lower bound, and we have taken the limit as *n *→ ∞, we can also estimate *δ*_∞_(*v_th_*) using (8) and indeed, using similar translation arguments, we estimate *δ*_∞_(*v*) = *δ*_∞ _for all *v *∈ [*v_r_*, *v_th_*].

Comparison of *γ_c _*(or ) and *δ*_∞ _suggests whether or not the critical kicks strategy will elicit more spikes than the other strategies we have described. If not, then we need additional arguments to assess the relative effectiveness of these alternative strategies. In fact, regarding alternative strategies, we have the following result:

**Proposition 1**. *The big kick strategy always yields at least as many spikes as the reset and kick (and equivalently, the threshold kick) strategy*.

*Proof*. The reset and kick strategy yields *m *+ 1 spikes, where *m *is the largest integer such that

Using equation (7), compute(9)

We can see from equation (9) that if *v *<*I*, then the slope *s *becomes more negative as *g *is increased, and if *v *>*I*, then the slope *s *becomes less negative as *g *is increased. Thus, the bands are narrowest at *v *= *I*; that is, *δ_n_*(*I*) < min{*δ_n_*(*v_r_*), *δ_n_*(*v_th_*) = *δ*_*n*+1_(*v_r_*)}. Hence, the big kick strategy, which elicits *m_b _*+ 1 spikes for the largest integer *m_b _*such that

always generates at least as many spikes as reset and kick.

### Band Width Estimation

In the previous subsection, we introduced a small number of intuitively reasonable strategies for eliciting the maximum number of spikes from model (2),(3) using a constrained input. We also used a phase plane approach to define a natural band structure, along with a corresponding idea of a band width *δ_n_*(*v*), which we used to show that two of these, the reset and kick and threshold kick strategies, will never be optimal. This structure can also be used to obtain an intuitive idea of which conditions favor a big kick strategy and which conditions favor the critical kicks strategy of giving many small kicks of the same particular size. Next, we use some approximations to derive additional quantitative information about *δ_n _*that can be used to determine more globally the optimal input strategy. Henceforth, in addition to assuming that *v_r _*= 0, we for convenience set *v_th _*= 1, with 0 <*I *< 1 <*E*.

We can estimate the magnitude *δ*(*g*) of the change in *g *that occurs over one spike cycle using the slope *s*(*v*, *g*) given in equation (7),(10)

where we have approximated *g *by a constant to estimate the integral. Note that for each *n*, the band widths *δ_n_*(0) = *δ*_*n*+1_(1) from the previous subsection are approximately equal to *δ*(*g*) for certain corresponding choices of *g*; for example, , where Γ_1 _intersects {*v *= 0} at . More generally, it is not necessary to choose a *g *associated with the boundary of a band, as defined from the previous subsection, in order to compute *δ*(g).

We can investigate the spikes of a cell by analyzing (10). This approach yields the following result.

**Proposition 2**. *If E *+ *I *- 2*EI *≥ 0, *then δ*(*g*) *is a monotone decreasing function of g*. *If E *+ *I *- 2*EI *< 0, *then δ*(*g*) *has a unique local minimum at a finite, positive value g *= *g*_0_.

*Proof*. Calculating the derivative of *δ*(*g*) with respect to *g*, we have

where

Furthermore,(11)

Equation (11) shows that *E *+ *I *- 2*EI *is indeed a key quantity.

Suppose now that *E *+ *I *- 2*EI *≥ 0. If *E *+ *I *- 2*EI *≥ 0, then *df*(*g*)/*dg *≥ 0, such that *f*(*g*) increases as *g *increases. Define

Since(12)

*f*_1 _increases as *E *increases. As

we have *f*_1 _≤ 0. Therefore, *f*(*g*) ≤ 0 for all *g *and hence *dδ*(*g*)/*dg *< 0. In another words, under the original approximation used to obtain (10), *δ*(*g*) decreases, and thus there is less change in *g *across each cycle from reset to threshold, as *g *increases.

Next, suppose that *E *+ *I *- 2*EI *< 0. Under this condition, *df*/*dg *changes signs, with *df*/*dg *> 0 for  and *df *(*g*)/*dg *< 0 for  From expression (12), we have *df*_1_/*dE *≤ 0 and lim inf_*E*→+∞ _*f*_1 _= 0, such that *f*_1 _≥ 0 for all *E *and *f*(*g*) ≥ 0 for . Thus, there exists a unique point *g*_0 _such that *f*(g) ≤ 0 for all  and *f*(*g*) ≥ 0 for all *g *∈ (*g*_0_,+∞). Correspondingly, *δ*(*g*) decreases when  and increases when *g *∈ (*g*_0_,+∞), where *g*_0 _is the zero point of *f*(*g*) = 0, and the proof is complete.

**Figure 2:**
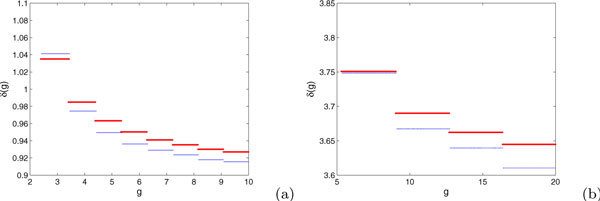
**The red lines display the estimate of *δ*(*g*) for (2),(3) obtained by our calculations, while the blue lines show *δ*(*g*) from direct simulation for *E *= 1.2, *I *= 0.7, and (a) *β *= 0.5, *G *= 10 or (b) *β *= 2, *G *= 20**.

**Figure 3:**
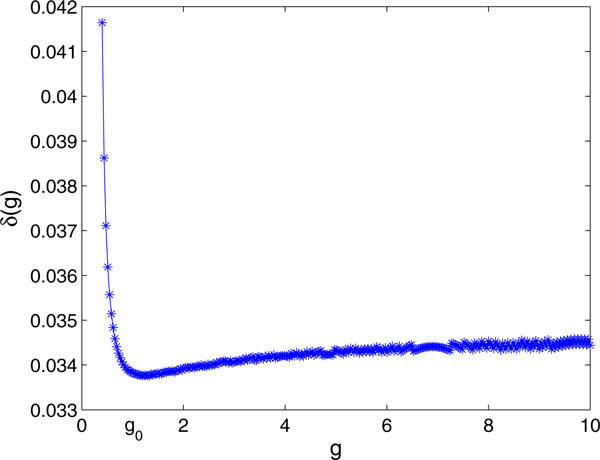
**A numerically calculated example of *δ*(*g*), with a unique local minimum, for parameter values *E *= 2, *I *= 0.7, *β *= 0.05, *G *= 10, such that *E *+ *I *- 2*EI *< 0**.

In the case of *E *+ *I *- 2*EI *≥ 0, the monotonicity of *δ*(*g*) suggests that for a trajectory evolving from an initial condition of (*v*, *g*) = (0, *g*(0)) to a final condition (*v*, *g*) = (1, *g*(*t*)), the drop *g*(0) - *g*(*t*) should be smaller for larger *g*(0). From Figure 2, we can see that, while the approximation used to derive equation (10) introduces an error in relative to the actual change in *g *computed from direct simulation of trajectories with *E *+ *I *- 2*EI *≥ 0, the error appears to be small and the monotonicity of *δ*(*g*) appears to be correct. Similarly, a numerically computed example of *δ*(*g*) for parameters that yield *E *+ *I *- 2*EI *< 0 is shown in Figure 3.

### Optimal Strategies and Spike Counts

Based on the previous two subsections, we conclude that if *E *+ *I *- 2*EI *≥ 0, then the loss of input with each spike, measured by *δ*(*g*), decreases as *g *increases and, furthermore, an input of a fixed size will cross the most spiking bands if it is given at *v *= *I*. Hence, of all possible strategies for eliciting spikes with an input of total size *G*, the one that yields the most spikes is what we earlier called the big kick strategy, unless *γ_c _*(or ) is sufficiently small that avoiding the bands altogether by following the critical kicks strategy is optimal. The optimal strategy when *E *+ *I *- 2*EI *< 0 is to provide a kick that puts *g *at approximately *g*_0_, the *g *value where the minimum of *δ*(*g*) occurs, and then provide as many kicks as possible of size *δ*(*g*_0_), again assuming that *γ_c_*,  are above a certain size. We will next perform some additional calculations that can provide estimates of numbers of spikes resulting from any strategy, which can be used with a minimum of calculation to compare the results of particular input sequences.

We first suppose that *E *+ *I *- 2*EI *< 0 and consider a generalization of the optimal strategy described above. That is, we assume that an initial input *G_i _*≥ *g*_0 _is given and then, once *g *evolves to some neighborhood of *g*_0_, kicks of size *δ*(*g*_0_) are repeatedly applied until the remaining input falls below *δ*(*g*_0_).

We now estimate the numbers of spikes Ω fired for each strategy of this type. Let Ω_1 _denote the number of spikes fired during the initial time period when *g *drops toward *g*_0_, let Ω_2 _denote the number of spikes fired during the final time period after the available input is depleted, and let Ω_3 _denote the number of spikes fired during the intervening period when repeated kicks of size *δ*(*g*_0_) are given. Clearly,(13)

and Ω = Ω_1 _+ Ω_2 _+ Ω_3_, so it remains to estimate Ω_1 _and Ω_2_.

Because of the shape of the function *δ*(*g*), the largest *δ*(*g*) during the initial time period will be associated with the first spike fired, while the largest during the final period will be associated with the last spike fired. We can estimate the drop *δ*(*G_i_*) in *g *up to the firing of the first spike from equation (10). To make this estimate relevant to other spikes early in the spike train, we take *v*(0) = 0 rather than *v*(0) = *I*.

Approximating the level of *g *in equation (10) by *g_i _*:= *G_i _*- *δ*(*G_i_*)/2, we obtain(14)

Next, we estimate *δ*(*g*) for the final spike fired, which we call *δ_f _*. To do this, we assume that when the final spike is fired, the trajectory reaches the lower bound on the *g *values that can yield a spike, namely the point of intersection of the *v*-nullcline and {*v *= 1}, at which . We also use the intermediate value of *g *across the trajectory Γ_0_(*v*), namely (1 - *I*)/(*E *- 1) + *δ_f _*/2, as the value of *g *for equation (10), which yields(15)

Now, to obtain an estimated spike count as *g *decays from *G_i _*to *g*_0_, we approximate *δ*(*g*) over each spike by the average of its two extreme values, (*δ*(*g*_0_) + *δ_i_*)/2. This approximation yields(16)

Similarly, once the input is used up, spikes continue to be fired as *g *decays from *g*_0 _to approximately (1 - *I*)/(*E *- 1), and the number of additional spikes that result is estimated by(17)

In the above equations, we have taken into account that the trajectory may not be reset precisely at *g*_0 _but rather somewhere within an interval approximated by (*g*_0 _- *δ*(*g*_0_)/2, *g*_0 _+ *δ*(*g*_0_)/2). Because this may lead to an overestimation by one or two spikes, we set . The total number of spikes fired is finally estimated by(18)

using equations (13)-(17).

The calculation can be easily generalized for input patterns that push *g *above and below *g*_0 _multiple times, although they will be non-optimal by our earlier arguments. Similarly, for an initial kick *G_i _*<*g*_0 _and *g *<*g*_0 _for all time, the smallest *δ*(*g*) available is *δ*(*G_i_*) and we can estimate the number of spikes resulting from partitioning the input into kicks of size *δ*(*G_i_*) by the equation(19)

If *E *+ *I *- 2*EI *≥ 0, then the same calculations still apply. The big kick strategy is optimal here, yielding a number of spikes estimated by(20)

Generalizing, a strategy of giving an initial input *G_i_*, followed by repeated kicks of size *δ_i _*until the input is depleted, yields a number of spikes estimated by(21)

**Figure 4:**
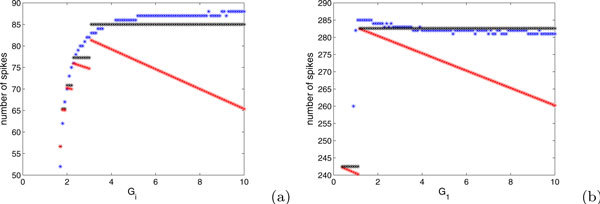
**Number of spikes fired as a function of *G_i _*for *G *= 100**. The blue points show results from direct numerical simulations of (2),(3) while the red and black points show the predictions from equations. Specifically, the red points are derived from (a) equation (21) with *E *= 1.2, *I *= 0.7 such that *E *+ *I *- 2*EI *> 0 and (b) equation (18) with *E *= 2, *I *= 0.7 such that *E *+ *I *- 2*EI *< 0. The same parameter values were used for the black points but the average (*δ*(*g_i_*) + *δ_f _*)/2 is replaced by *δ*(*g_i_*) in equation (21) in (a) and the average (*δ*(*g*_0_) + *δ*(*g_i_*))/2 is replaced by *δ*(*g*_0_) in equation (16) in (b).

Figure 4 shows comparisons of our spike estimates from equations (18) and (21) with numerical computed counts of spikes, illustrating that our estimates can be reasonable.

In fact, in the case of *E *+ *I *- *2EI *≥ 0, we underestimate the number of spikes fired for large *G *and *G_i_*. This underestimation results because we average *δ*(*G*) or *δ_i _*with *δ_f _*in the denominator of equation (20) or (21), whereas most spikes yield decreases in *g *that are much smaller than *δ_f _*. Improved estimates in such cases be obtained by weighting this denominator more toward *δ*(*G*) or *δ_i_*, which will decrease the denominator and thus will always yield predictions of additional spikes for larger kicks, relative to the formulas in equations (20),(21). An example resulting from extreme weighting, replacing the average of *δ_i _*and *δ_f _*with *δ_i _*alone, is also shown in Figure 4, as is a similar example for the case of *E *+ *I *- 2*EI *< 0. In summary, equations of the form (18)-(21), each requiring calculation of only a small number of quantities, can be used on a case by case basis to estimate the numbers of spikes that will result from a given strategy and therefore to compare strategies. These formulas provide for an informed comparison between the two types of big kick strategies determined to be optimal for the two distinct cases of *E *+ *I *- 2*EI *≥ 0 and *E *+ *I *- 2*EI *< 0, respectively, and the critical kicks strategy. Furthermore, now that we have defined *δ*(*g*), we can give a more precise variation on the calculation of equation (9) made in the subsection on phase plane structures and basic strategies to show that truly optimal strategies (other than the critical kick strategy based on ) provide kicks with *v *= *I*, so each strategy should include a time shift so that kicks are given when this condition is met, rather than with *v *= 0 or *v *= 1. Specifically, if *g*_1 _>*g*_2_, then for *v*_0 _∈ 0[1],

Calculating the derivative of the above equation with respect to *v*_0 _yields

a quantity that is positive for *v*_0 _<*I *and negative for *v*_0 _>*I*. Hence, the additional input needed to cross bands is minimal for kicks given at *v*_0 _= *I*, in agreement with Proposition 1. Finally, it is not difficult to see from examination of the above spike counts and equation (10) that, with other parameters fixed, increases in *β *yield fewer spikes, as expected from the corresponding faster decay of *g*, while increases in *E *and *I *yield more spikes, as expected from the increased rate of change of *v*.

## Theta Model with Synaptic Kicks

### Model

We next consider a theta model neuron receiving positive synaptic excitatory kicks, governed by the equations(22)

where *b *< 0 is a parameter. We consider *θ *∈ [-*π*, *π*] mod 2*π*, and the neuron is said to fire when *θ *increases through *π *and is effectively reset to -*π*. With *g *= 0, corresponding to the absence of excitatory inputs, and *b *< 0, which is the case we consider, the theta model (22) has two critical points, namely a stable fixed point at  and an unstable fixed point at . Moreover, we assume that the arrival of, and constraints on, synaptic excitation are identical to those for the LIF model, given by (5), (6), with *G *> 0 fixed. Note that, as in the LIF case, everything that we do in this section would still apply if we chose *b *sufficiently large that the model fired spikes in the absence of input, but we stick with the *b *< 0 case to include the additional effects associated with the requirement of a minimal input for spiking to occur.

### Existence of an optimal *g*

We proceed by approximating the amount by which *g *decreases as a trajectory evolves from (-*π*, *g_i_*) to (*π*, *g_f_*), analogously to our calculations in Section 2. Fixing *g *at some value between *g_i _*and *g_f _*, we have(23)

A straightforward calculation yields the following result.

**Proposition 3**. *The function δ(g) defined in (23) has a unique local minimum at g *= -2*b*.

*Proof*. We calculate

So *dδ*(*g*)/*dg *< 0 when *g *< -*2b*, and *dδ*(*g*)/*dg *> 0 when *g *> -2*b*. Clearly, *δ*(*g*) has the minimum value .

**Figure 5:**
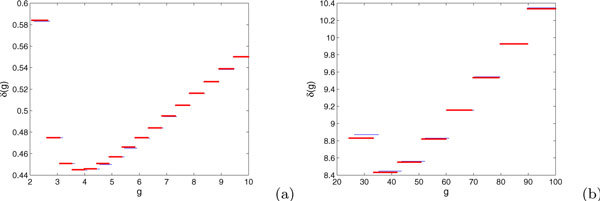
**The red lines represent *δ*(*g*) for the theta model from equation (23), while the blue lines give the size of the change in *g *observed from direct simulation of system (22) for (a) *b *= -2, *β *= 0.05, *G *= 10 and (b) *b *= -20, *β *= 0.3, *G *= 100**.

In Figure 5, we validate the approximation of holding *g *constant in equation (23) by showing that such a minimum exists in direct simulations of the theta model.

### Optimal Strategies and Spike Counts

**Figure 6:**
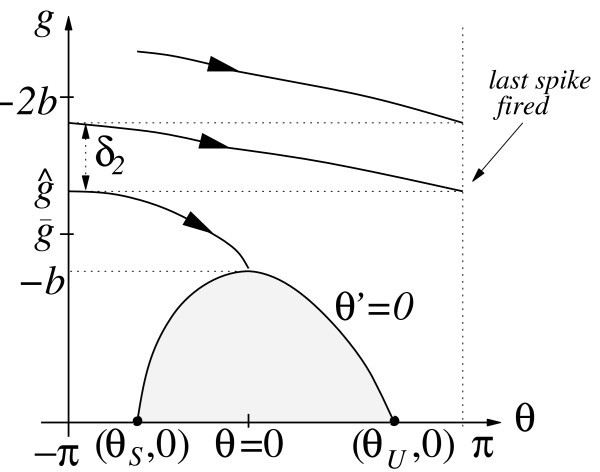
**Schematic illustration of the (*θ*, *g*) phase plane including our approximation to  and .** The shaded region indicates the set where *θ' *< 0. *δ*_2 _is the change in *g *as *θ *evolves from -π to π for the firing of the neuron's final spike.

We will now estimate the number of spikes that will result from a given input allocation strategy. To make effective estimates, it is helpful to estimate the minimal value, call it , such that a trajectory starting from (-*π*, *g*) will result in a spike if and only if . To do this analytically, we seek  such that the trajectory from  reaches (0, -*b*) and thus crosses the *θ*-nullcline and converges to *θ_S_*; see Figure 6. Although there are other trajectories with initial *g *values above this  that also converge to *θ_S _*, instead of spiking, by crossing the *θ*-nullcline at points with *θ *> 0 and *g *< -*b*, this approach nonetheless gives a reasonable approximation to the true value of .

We again approximate *g *as a constant over the trajectory of interest, namely , which is the average of the initial value of *g *and the value of *g *at *θ *= 0 (Figure 6). This approximation yields

and thus

which provides an implicit estimate for .

Now, if , then all inputs that yield spikes must push the trajectory to *g *values for which *dδ*(*g*)/*dg *> 0 holds. Thus, pushing *g *to progressively larger values yields fewer spikes, and a critical kicks strategy, with initial kick size  and subsequent kick sizes , is optimal, yielding approximately(24)

spikes.

If , then there is a tradeoff: investing more input initially, up to about size -2*b*, will push the trajectory to a region where *δ*(*g*) is minimal. On the other hand, if less input is initially invested, then there is more input remaining to give subsequent kicks. We ignore strategies in which an initial input  is given, some nonzero number of spikes is fired, and then an additional large input spanning multiple spiking bands is given, since these can be seen always to be non-optimal. Suppose first that the initial input has a size *G_i _*< -2*b*. Given the shape of *δ*(*g*), the optimal strategy is to expend the remaining input on(25)

kicks of size *δ*(*G_i_*), analogously to the strategy behind equation (24). Which *G_i _*< -2*b *is optimal depends on the sizes of ,*b*, and *δ*(*g*).

Alternatively, the other possible optimal strategy, if *G *> -2*b*, is to take *G_i _*≥ -2*b *and to try to maintain *g *values close to *g *= -2*b*. For an initial input *G_i _*> -2*b*, we estimate the number of spikes for such a strategy using a similar approach to that used in subsection 2.4, breaking up the estimate into an initial period of decay of *g*, a period of kicks to keep *g *near -2*b*, and a final period of spiking until *g *drops below . Once the initial input is given, *δ*(*g*) for the first spike is approximated by a solution to the equation

We will also use an estimate of the *δ*(*g*) for the last spike fired (Figure 6), obtained from the equation

Now, for , recall that *dδ*(*g*)/*dg *< 0 when *g *< -2*b *and *dδ*(*g*)/*dg *> 0 when *g *> -2*b*. Thus, during the period when *g *> -2*b*, the largest *δ*(*g*) is given by *δ*_1_, and while *g *< -2*b*, the largest *δ*(*g*) is *δ*_2_. The smallest *δ*(*g*) is of course *δ*(-2*b*). Following our earlier strategy of estimating *δ*(*g*) by the average of its largest and smallest values, and approximating *g *over one spike interval by the initial value minus half of the drop in *g *that occurs during that interval, we obtain our estimated spike counts. Specifically, during the initial period of input decay from *G_i _*to approximately -2*b*, our estimate is

During the final period of input decay from approximately -2*b *to approximately , our estimate is

Finally, the number of repeated spikes from the critical kicks of size *δ*(-2*b*) given until the remaining available input *G *- *G_i _*is depleted is about

Given that Ω_1 _and Ω_2 _could each overestimate the number of spikes by one, we estimate the total number of spikes with input *G *as(26)

A similar estimate can be obtained from other patterns of inputs.

**Figure 7:**
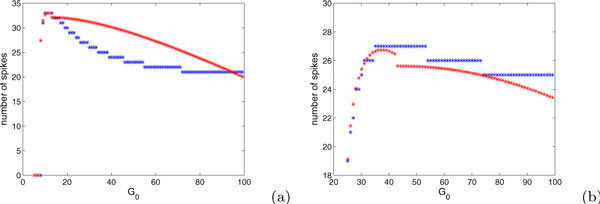
**Spike counts for the theta model (22)**. The red curve denotes the number of spikes estimated from equations (25),(26), while the blue lines show numbers of spikes observed in direct simulations of the theta model with *G *= 100 and (a) *b *= -5, *β *= 0.2; (b) *b *= -20, *β *= 0.1. For the simulations, an initial kick of size *G_i _*was given. If *G_i _*≥ -2*b*, then the trajectory was allowed to decay until *g *≈ -2*b *- *δ*(-2*b*) and then the remaining input was expended on kicks of size *δ*(-2*b*). If *G_i _*< -2*b*, then after an initial spike was fired, the remaining input was expended on kicks of size *δ*(*G_i_*). These same strategies were assumed for the analytical estimation.

In summary, we have two candidate optimal strategies when . Depending on the relative sizes of  and -2*b *and the shape of *δ*(*g*), it may be optimal to choose initial input *G_i _*< -2*b *and give repeated kicks of size *δ*(*G_i_*) or it may be optimal to choose *G_i _*≈ -2*b *+ *δ*(-2*b*)/2 and provide repeated kicks of size *δ*(-2*b*), in both cases repeating the kicks until the input is depleted. Figure 7 shows examples illustrating that the optimal *G_i _*is indeed very close to -2*b*.

We can extend this analysis one step further by determining the best value of *θ *at which to deliver the input kicks.

**Proposition 4**. *Given an initial condition (-π, g) and an input of a fixed size, the maximal number of spikes subsequent to input delivery is attained when these inputs are given with *.

*Proof*. Suppose *g*_1 _>*g*_2_. We have

Calculating the derivative of the above equation with respect to *θ*_0_, we have

As *d*(*δ*(*g*_1_, *θ*_0_) - *δ*(*g*_2_, *θ*_0_))/*dθ*_0 _> 0 for  and *d*(*δ*(*g*_1_, *v*_0_) - *δ*(*g*_2_, *v*_0_))/*dv*_0 _< 0 for , the difference *δ*(*g*_1_,*θ*_0_) - *δ*(*g*_2_, *θ*_0_) will be maximal at  and minimal at . Hence, a maximal number of spikes is attained from an input given with .

## Theta Model with Continuous Input

In the analysis we have done so far, the input to the neuron arrives as a series of discrete kicks. An excitatory postsynaptic potential evoked by an individual input may have a more gradual rise, however. We now switch gears and consider how such an individual input, arriving with a continuous time course, can be optimally tailored to evoke a maximal response. This analysis is also relevant to a situation in which a neuron receives input from a very large presynaptic population that fires in near synchrony, but with some spread in recruitment times.

The theta model with a continuous input can be described by the equations(27)

with

where *b *< 0 and *A*, *β *> 0 are parameters. The form of *γ*(*t*) in equation (28) is often used computationally and has been specifically selected to ensure that its integral over the positive time axis is fixed at *A *for all values of *β*. Unlike the case where the *θ *model received synaptic kicks, we now consider *θ *∈ **R**, with a spike fired whenever *θ *increases through *nπ *for an integer *n*.

The question that we now address is, given a fixed set of intrinsic parameters *b *and *A *and fixed *P *> 0, how should *β *be selected to yield the maximum number of spikes within the time interval [0, *P*]? Note that when *θ *= *nπ*, *θ' *> 0, so it suffices to find *β *to maximize *θ*(*P*).

### Boundary Value Problem

We will find optimal values of *β *by numerically solving a boundary value problem. For numerical purposes, it is convenient to map *t *∈ [0, *P*] to *s *∈ 0[1] using *s *= *t*/*P*. Correspondingly, let denote *d*/*ds*, such that equation (27) becomes(29)

Next, differentiate  with respect to *β *to obtain(30)

where  is given by(31)

To the *θ *and *θ_β _*equations, we append the additional equations(32)(33)

Given the system of four equations (29),(30),(32),(33), we need a set of four boundary conditions. First, we set *θ*(0) = *θ_S _*= - arccos((1 + *b*)/(1 - *b*)), so that the model neuron is at a stable rest state when input starts to arrive. Since this specification is independent of *β*, we have *θ_β_*(0) = 0. To find an extreme (with respect to *β*) value of *θ*(*P*), we set *θ_β_*(*P*) = 0 as well. Finally, we take *t*(0) = 0. We solve this boundary value problem (BVP) numerically, using XPPAUT [15], to obtain the optimum *β *for any fixed *P*, *A*, *b*.

### Results

**Figure 8:**
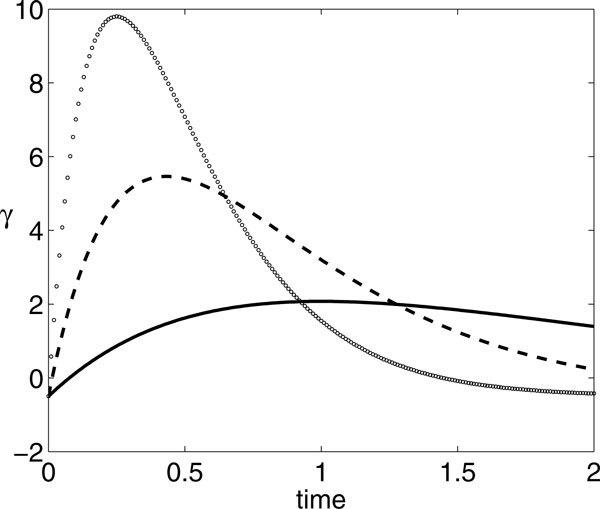
***γ*(*t*) for *A *= 7, *P *= 2, and *β *= 1 (solid), 2.316 (dashed), and 4 (dotted)**.

**Figure 9:**
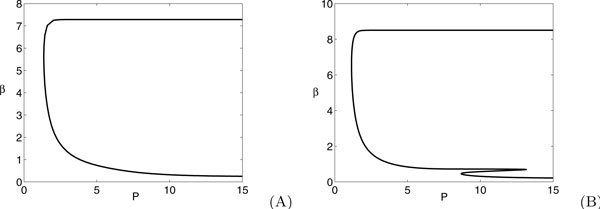
**Optimal *β *versus *P *for (A) *A *= 7 and (B) *A *= 8**.

**Figure 10:**
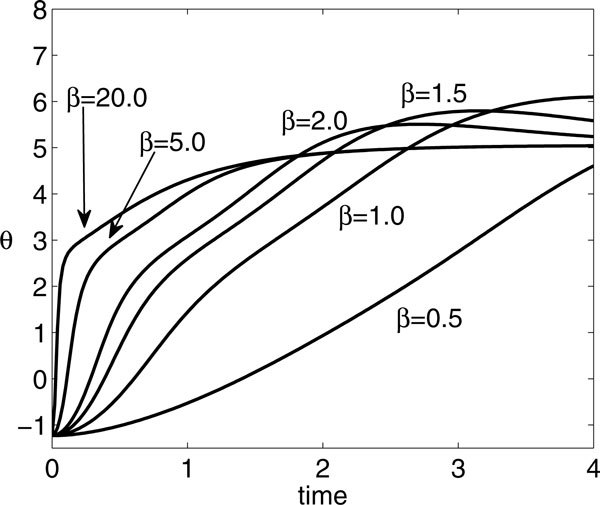
**Time course of *θ *for *A *= 7, *P *= 4, and various *β *values as labeled**. Based on maximizing the value of *θ*(4), the extremal *β *for *A *= 7 and *P *= 4, as plotted in Figure 9A, are *β *≈ 0.95 (lower branch) and *β *≈ 7.28 (upper branch); however, *θ*(4) appears to be identical (*θ *≈ 5.04) for all *β *sufficiently large.

If *A *and *P *are fixed, then varying *β *yields different shaped input functions *γ*(*t*), as illustrated in Figure 8. For fixed *A*, if *β *is sufficiently large, then the input is sufficiently concentrated in time that *P *becomes irrelevant for the number of spikes fired. Alternatively, for smaller *β*, input arrives more gradually and *P *becomes relevant. These relationships are evident in Figure 9A, which shows a curve of solutions to the optimization BVP described in the previous subsection, plotted in *β *versus *P *space. Direct numerical simulations show that in fact the same *θ*(*P*) arises for other large *β *as occurs for the *β *value along the upper branch of the curve in Figure 9A, as long as *P *is sufficiently large that this curve is flat (Figure 10). Along the rest of the curve, the *β *values shown truly represent local extrema. In particular, the lower branch of the curve represents local maxima for *θ*(*P*). For *P *values such that the upper branch is flat, the lower branch appears to actually consist of global maxima (Figure 10), while the upper branch represents local minima for sufficiently small *P *that this this branch has curvature (e.g. *P *near 2), and for these *P*, there may be additional maxima for *θ*(*P*) at large *β*, not shown in Figure 9A.

**Figure 11:**
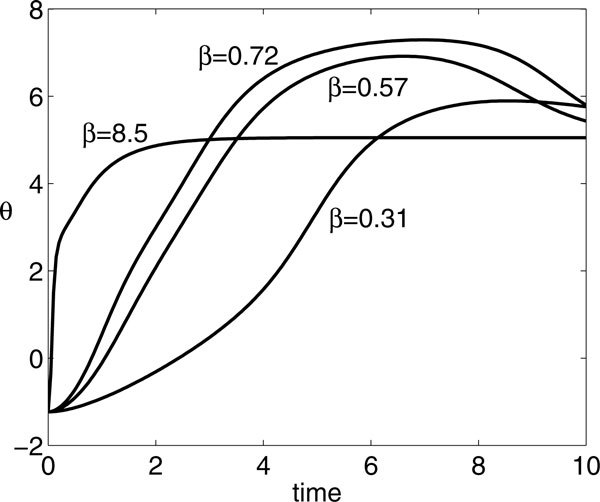
**Time course of *θ *for *A *= 8, *P *= 10, and various *β *values as labeled, which yield extremal values of *θ*(10)**.

**Figure 12:**
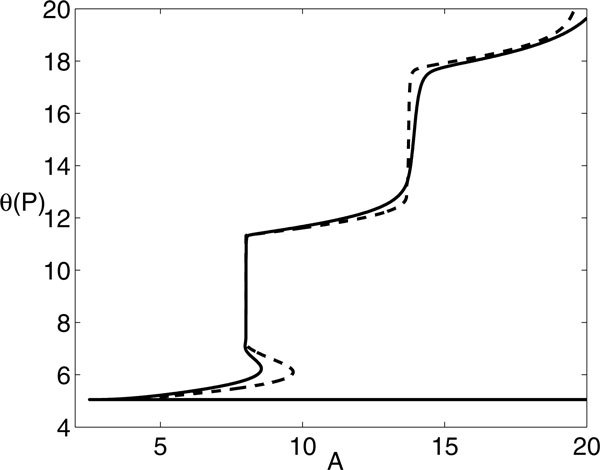
***θ*(*P *) versus *A *for *P *= 10.5 (solid) and *P *= 13 (dashed), along curves of extrema obtained by solving the BVP**. The two curves coincide on the low, horizontal branch near *θ*(*P*) = 5. For each *P*, note the existence of multiple extrema for a small interval of *A *values as well as the occasional abrupt increases in *θ*, corresponding to spike addition.

**Figure 13:**
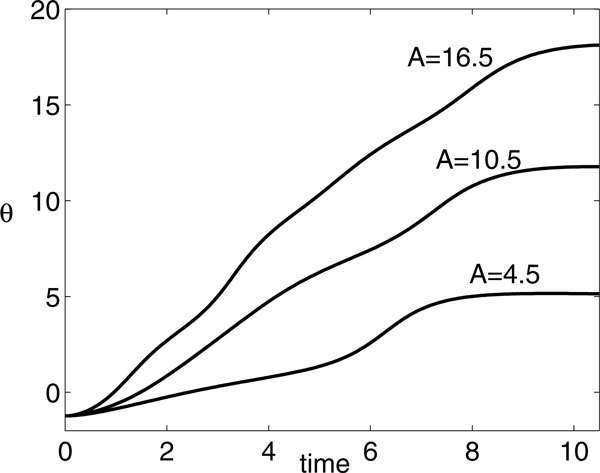
**Time courses of *θ *for optimal values of *β *found for *P *= 10.5 and various *A *values as labeled**. Note that *θ*(10.5) increases by slightly more than 2*π *as *A *increases from 4.5, with *θ*(4.5) ≈ 5.052, to 10.5, with *θ*(10.5) ≈ 11.771, and from 10.5 to 16.5, with *θ*(16.5) ≈ 18.123, corresponding to the abrupt jumps in Figure 12.

For other values of *A*, the situation is similar to Figure 9A; however, some subtleties do emerge. As shown in Figure 9B, additional local extrema of *θ*(*P*) may arise at small *β*, yielding an interval of *P *values with three such extrema at small *β*, bracketed by two fold bifurcations. For example, for *A *= 8 and *P *= 10, there are a local maximum near *β *= 0.31, a local minimum near *β *= 0.57, and another local maximum near *β *= 0.72, with corresponding *θ*(*t*) shown in Figure 11. Note that, as with *A *= 7 as well as other values of *A*, *θ*(*P*) saturates for sufficiently large *β*. The folding structure in Figure 9B arises only for a small interval of *A *values that is difficult to pin down precisely. It is also illustrative to view what happens to the families of BVP solutions as *A *is varied with *P *fixed. Of course, the multiple extrema show up here as well, as evidenced in Figure 12, along with some additional nontrivial dependence of the optimal *β *on *A*. By comparing these bifurcation curves for different *P *, we see that the interval of multiple extrema moves to larger *A *as *P *increases (and vice versa). Furthermore, as *A *increases for fixed *P *, *θ*(*P*) at optimal *β *can undergo abrupt increases, associated with the firing of an additional spike (Figure 13); the interesting structure of the BVP solution curves appears to be related to these events.

## LIF Model with Continuous Input

With the continuous input *γ*(*t*) given in equation (28), we used variational methods to find *β *values that yielded extremal values of *θ*(*P*) for the *θ *model (27), given a final time *P *> 0. Such methods are not available for the LIF model due to the discontinuity imposed by its reset condition. Nonetheless, we can perform some direct analysis of the dependence of firing in the LIF model on the parameter *β*. In particular, although we will not specify an optimal *β*, we will analytically establish some results about how spike times depend on *β*, including the fact that the number of spikes saturates as *β *grows, which is consistent with [13] and contrasts with the non-monotone dependence of *θ*(*P*) on *β *seen in the previous section. Similar methods can be applied to attain analytical results for the theta model, and these are briefly discussed in the Appendix; in particular, these show that spiking is lost when *β *becomes sufficiently large.

To perform this analysis for the LIF model, we make a fairly strong approximation. If a spike is fired at time *T_a _*and the next spike occurs at time *T_b_*, then on the time interval (*T_a_*, *T_b_*], we approximate *γ*(*t*) by the time average(34)

with(35)

Note that by definition,  and *v*(*T_b_*) = 1, such that(36)

If we assume that *v*(0) = 0 and fix a positive integer *n*, then we can try to solve equations (34) and (36) with *T_a _*= 0 for a pair of positive numbers that we label , such that the solution of equation (35) satisfies *v*(*T*_1_) = 1. Similarly, we can set *T_a _*= *T*_1 _and solve for , and inductively, given , we can solve for  until we find *T*_*n*+1 _>*P *for some *n*. At that point, we would propose that *T*_1_, ..., *T_n _*are our approximate spike times in the interval [0, *P *]. In practice, to constrain the solution set of this system of equations, we assume that *T_n _*= *P *, since numerical explorations show that, for fixed *A *and *P*, if *β *is tuned to maximize the number of spikes generated on *t *∈ [0, *P *] by the LIF model with input given by equation (28), the final spike time is indeed generally close to *P*. Thus, for fixed *A*, *β*, *P*, instead of solving iteratively, we attain candidate approximate spike times by simultaneously solving *n *copies of equation (34) and *n *copies of equation (36) for unknowns  with a final spike time *T_n _*= *P *.

We next study constraints on these approximate spike times {*T_1_*, ..., *T_n_*}. Specifically, we have the following result.

**Proposition 5**. *For fixed A, P, there exists β_1 _>*0 *such that if β < β*_1_*, then there are no spikes (as defined above). If β > β*_1_*, then there exist  such that all spikes lie in . The function t(β) is monotone decreasing in β and is bounded above by *1*/β. There also exists β_2 _> β_1 _such that the function  is monotone increasing for β *∈ (*β*_1_*, β*_2_)*, achieves its maximum at β *= *β*_2_*, and is monotone decreasing with  for β > β*_2_*. The value of β*_2 _*is given by the minimal positive solution of *.

*Proof*. First, note that spikes can only occur if . Clearly, the equation(37)

has , on the line *βt *= 1, as one solution. A brief calculation verifies that equation (37) has no solutions for *β *<*β*_1_, while for *β *>*β*_1_, there are two solutions, (*β*, *t*(*β*)) in {*βt *< 1} and  in {*βt *> 1}. For fixed *β*, all spikes must lie in the time interval ; note that, in particular, *T*_1 _is bounded below by *t*(*β*), while our assumption that *T_n _*≈ *P *would limit us to parameter choices such that , although this inequality need not be satisfied for this proposition to hold.

Now, consider a solution *t*(*β*) of equation (37). Differentiation of (37) yields(38)

while equation (38) itself yields(39)

Equations (38),(39) imply that *t*(*β*), *βt*(*β*) are monotone decreasing, since(40)

for all *β *for which it is defined. Initially, equation (38) also implies that  is increasing, since the curve  lies in 1 <*βt *< 2 for *β *near *β*_1_. However,  also increases, by equation (39), until  achieves a maximum at *β*_2 _such that . For *β *>*β*_2_,  decreases, by equation (38), but  for all *β *>*β*_2_, since the curve *βt *= 2 has a negative slope in the (*β*, *t*) plane and  at *βt *= 2.

**Figure 14:**
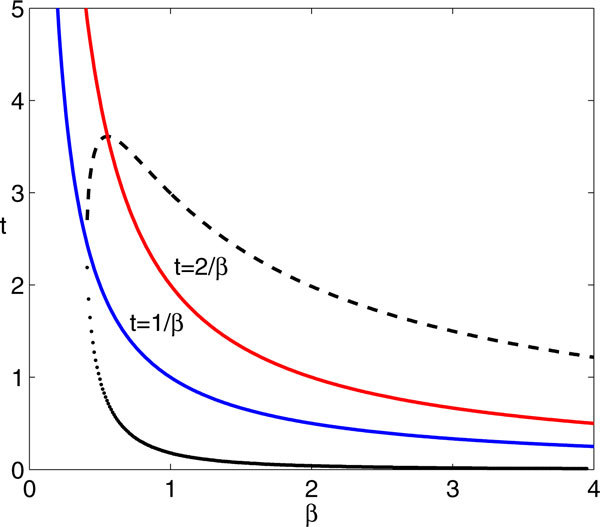
**Plot of the solutions to equation (37) for *A *= 10, *E *= 1.2, *I *= 0.7**. The curves shown are *t *= 2/*β *(red), *t *= 1/*β *(blue),  (dashed black), and *t *= *t*(*β*) (large dotted black). Note that *t*(*β*) < 1/*β *and that  rises up from *t *= 1/*β *to its maximum at *t *= 2/*β *and then decays, in agreement with Proposition 5.

**Figure 15:**
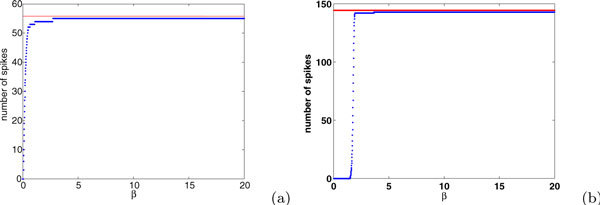
**Number of spikes generated by the LIF model with *A *= 100, *p *= 10 and (a) *E *= 1.2, *I *= 0.7; (b) *E *= 2, *I *= 0.3**. The blue curves show results of direct simulations of the LIF model with continuous input *γ*(t) while the red lines are at *A*/ln(*E*/(*E *- 1)).

An example of the solution curves to equation (37), illustrating the results of Proposition 5, appears in Figure 14. The curves of possible spike times *T_i_*(*β*) lie between *t*(*β*) and  described above. Of course, if *P *is small, the spiking that can occur in time [0, *P *] will be limited. With large *P*, so that the full collection of predicted spike times is realized, direct simulations suggest that the number of spikes increases with *β *(e.g., Figure 15). While we will not prove that result, we can establish some properties of spike times in the model in the limit of large *β*:

**Proposition 6**. *As β *→ ∞,

*where n is the total number of spikes fired*.

*Proof*. The first limit is an immediate consequence of the bound (40). The second limit follows from equation (37). Specifically, some algebra yields

with the left hand side clearly converging to 0 as *β *→ ∞. Moreover, the inequality

yields

so  as *β *→ ∞.

It remains to establish the fact that number of spikes converges as *β *becomes sufficiently large, as stated in the Proposition and seen in Figure 15. First, note that the difference between successive spike times goes to 0 as *β *→ ∞ since  in this limit. Thus, the left hand side of equation (36) tends to 0 as *β *→ ∞.

Maintenance of the equality in equation (36) therefore requires that(41)

which is consistent with the fact that the maximum of *γ*(*t*) blows up with *β*.

Combining equations (34) and (36) yields the equality

and equation (41) implies that the limit of the right hand side equals ln(*E*/(*E *- 1)). Hence, we define a constant *C*_1 _satisfying(42)

A solution of equations (34), (36), with *T*_0 _= 0, is *T*_1 _= *C*_1_/*β*, . Next, define a constant *C*_2 _satisfying(43)

Addition of equations (42), (43), after rearrangement, yields

and *T*_1 _= *C*_1_/*β*, *T*_2 _= (*C*_1 _+ *C*_2_)/*β*,

is another solution of equations (34), (36).

**Figure 16:**
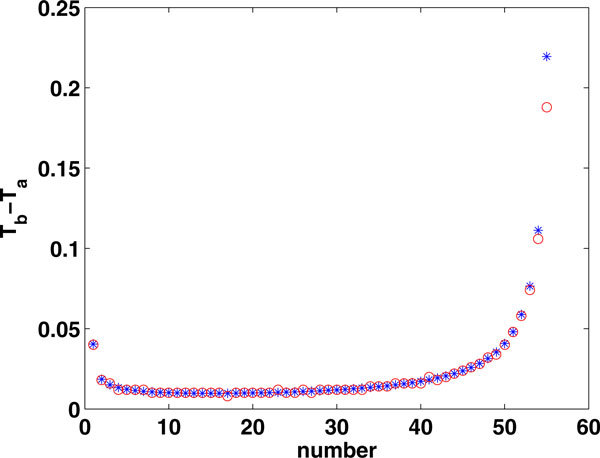
**Times between spikes fired by the LIF model with *A *= 100, *p *= 10, *β *= 5, *E *= 1.2, *I *= 0.7**. The blue symbols show the results of direct simulations of the LIF model with continuous input *γ*(t) while the red circles show solutions of equations (34), (36) obtained using the serial procedure described in the text.

Repeating this process, we can find a series of solutions to these equations (see Figure 16), such that for the *j*th spike, ,

and(44)

We denote the total number of spikes by *n*(*β*) and estimate the final spike time *T*_*n*(*β*) _for any fixed *β *by , which gives  as *β *→ ∞. Thus, from equation (44), it follows that lim_*β*→∞ _*A *- *n*(*β*) ln(*E*/(*E *- 1)) = 0, which yields

as desired (Figure 15).

## Discussion

### Summary and modeling issues

We have considered how certain constrained, positive inputs should be timed to yield maximal numbers of spikes in the LIF and theta models for neurons. In both models, we have considered parameter regimes in which inputs must be above a threshold to elicit a spike. Thus, when each model is subjected to a train of discrete inputs of a fixed total magnitude, it is possible that maximal firing is attained by a critical kick strategy of giving just enough input to push the model trajectory above this threshold and then giving the minimal kick after each spike that achieves threshold clearance again. Aside from this possibility, we have analytically identified which other strategies could possibly be optimal for each model. Defining *δ*(*g*) as the magnitude of the change in input from the level *g *at the firing of one spike to the level *g *- *δ*(*g*) at the firing of the next spike, we found for the LIF model that our analytical approximation to *δ*(*g*) could be monotone decreasing in *g *or not, depending on the sign of *E *+ *I *- 2*EI*. In each case, we present an optimal strategy. If *E *+ *I *- 2*EI *≥ 0, the optimum is a big kick strategy in which all available input is provided immediately. If *E *+ *I *- 2*EI *< 0, if the minimum of *δ*(*g*) occurs at *g *= *g*_0_, and if the amount of input available exceeds *g*_0_, then the optimum consists of an initial kick of size ≈ *g*_0_, followed by kicks of size ≈ *δ*(*g*_0_) after each spike, until the input is depleted. This dichotomy of possible outcomes may be unexpected in light of standard intuition about the LIF model, which emphasizes the power of big, synchronized input kicks to induce firing. Furthermore, the definition and analysis of *δ*(*g*) is itself novel, and its non-monotonicity in *g *means that there is a different effective dissipation of inputs at different input strengths, defined relative to the time it takes to progress from repolarization after a spike to firing of the next spike. This idea, that the power of a synaptic input depends on more than the individual input's amplitude, decay rate, and arrival phase, is often neglected in neuroscience studies and is an important observation of our work.

Unlike the LIF model, our approximation to *δ*(*g*) always has a unique local minimum for the theta model, and we can compute its location directly. Assuming that this minimum corresponds to an initial condition that really does result in a spike, there is a clear optimal strategy of providing critical kicks to keep *g *near the minimum of *δ*(*g*), as in the LIF case with *E *+ *I *- 2*EI *< 0. The existence of this minimum likely is related to the presence of an internal peak in the phase response curve (PRC) for the theta model, which past authors have suggested would represent an optimal phase for input timing [16], although PRC theory applies to oscillators receiving weak inputs, while we consider strong inputs in the excitable regime.

Heuristically, this minimum may arise because of the shape of the *θ*-nullcline in the (*θ*, *g*) plane. If *g *is on the small end of the spiking range, then the progress of *θ *towards spike threshold is slowed by the proximity of the trajectory to the *θ*-nullcline, allowing *g *to drop significantly from one spike to the next. If this idea is correct, then we expect non-monotone *δ*(*g*) to occur for models where voltage can be significantly slowed between spikes, without preventing the firing of the next spike, when *g *is on the lower end of the spiking range. In summary, for both the LIF and theta models with discrete inputs, the maximal number of spikes is attained for one of two strategies, either a critical kick strategy based on a minimal input threshold or a second big kick or critical kick strategy that we have identified from analysis of *δ*(*g*). The latter applies equally well in the case when the models are intrinsically oscillatory in the absence of inputs.

We also show how to estimate analytically the number of spikes resulting from any input time course in both models, obtaining results that compare well with direct numerical simulations. Further, we establish an optimum value for the dependent variable of each model, *v *and *θ *respectively, where each input should be delivered in a critical kick strategy to achieve maximal subsequent spike output. In both cases, this value is a stable critical point for the intrinsic dynamics of the model.

When the input to each model is continuous, the optimization problem we consider is how to adjust the shape of the input, using a parameter *β *that does not affect the input's overall magnitude, to achieve maximal spiking in a fixed time interval [0, *P *]. Derivation and solution of a BVP yields values of *β *eliciting extremal numbers of spikes for the theta model, and numerical simulations show whether these extrema are maxima or minima. We find that the number of spikes fired increases and then decreases again as *β *increases, corresponding to faster rise and decay of the input function and a larger maximal input. The existence of an interior local optimum for *β *is consistent with the non-monotonicity of *δ*(*g*) for the theta model in the discrete input case, with both observations pointing out that delivery of input in a stronger, faster way may reduce the resulting number of spikes for the theta model. Eventually, the number of spikes saturates, remaining invariant under additional increases in *β*.

The LIF model with a continuous input shares this saturation property, as we prove in Proposition 6. For the LIF model, however, unlike the theta model, the number of spikes increases monotonically with *β*, and hence with the degree to which the input is concentrated in time, based on numerical simulations. This finding is consistent with previous analysis [12,13] showing that synchronous input to the LIF model yields more spiking than inputs that are spread out. Indeed, in light of this set of results, it is interesting that we do not always find this behavior for the LIF model with discrete input kicks, in the case where *E *+ *I *- 2*EI *< 0. This disparity in results for the LIF model points out that details of how synaptic inputs are modeled can influence model dynamics in significant ways. The specific differences between optimal input patterns for the LIF and theta models that we highlight represent novel findings about the relationship between these models, while other differences have been demonstrated in earlier work [12]. Both of these models have Type I behavior [10,17], including the ability to fire spikes at arbitrarily low frequencies, yet clearly there are differences in their dynamic properties, including their responses to inputs. Such subtleties point out that classifications of models and neurons into gross categories, such as integrators and resonators, often need additional refinements to capture the diversity of neuron dynamics.

Analytically, we found an interval of times during which all spikes must occur for the LIF model with continuous inputs, and we characterized the interesting dependence of the endpoints of this interval on *β*. The results of applying similar methods to the theta model are also discussed in the Appendix. As *β *increases, all spike times converge to 0, yet the input becomes strong enough to elicit increasingly more spikes (up to some level). We were not able to exploit our approach to prove that the number of spikes increases monotonically with *β*, however. More specifically, we identified a minimal value *β*_1 _such that *β *>*β*_1 _is necessary for spikes to occur. One idea for proving monotonicity would be to seek a sequence of values *β*_1 _<*β*_2 _<*β*_3 _< ... such that *β *>*β_n _*is required for the firing *n *spikes (note that this *β*_2 _differs from the *β*_2 _used in Section 5). However, we were able to derive an equation for *β*_1 _because we have an analytical formula for a minimal level of input needed for at least one spike to occur, and we do not have such an expression for subsequent spikes. Nonetheless, it is possible that the band structure established in Section 2 could be exploited to abstractly establish results in this direction that might lead to a proof of monotonicity.

The overall utility of the techniques presented in this paper depends in part on their generalizability. Note that the models we consider have relatively few parameters, and our results do not depend on particular parameter choices as long as they render the neuron excitable. For our numerical examples, we have chosen various parameter combinations to try to illustrate different parameter regimes. In some discrete input cases, we did choose *β *values that appear to be too small to represent fast AMPA-mediated synaptic excitation, because certain differences in outcomes across strategies are most clearly evident with small *β*. It is important to keep in mind, however, that our models are dimensionless, that there are also slower NMDA-mediated excitatory synaptic currents, and that what we have represented as a slow synaptic decay could also arise from a long membrane time constant in a postsynaptic neuron or from the gradual arrival of many inputs from a presynaptic neuron population with a slowly down-ramping firing rate. Aside from parameter variations, our methods for estimating numbers of spikes are also quite general across different discrete input patterns, for the models we consider. For these models, our methods could likely be adapted to other optimization problems, such as tuning inputs to achieve regularity of interspike intervals, spiking within some range of rates, or spiking at particular times [7] or finding minimal inputs to generate particular spike patterns [8]. In each of these problems, we would obtain predictions about which features of the timing and size of inputs within a discrete input train yield which spiking patterns.

We have assumed that the neurons receiving inputs are silent in the absence of inputs. As noted above, our techniques for the discrete input case would generalize to models with nonzero intrinsic firing rates, and in particular the band structure partitioning phase space into initial conditions corresponding to different numbers of spikes fired before inputs are depleted would carry over analogously. Indeed, the existence of a minimal level of input needed for spiking becomes irrelevant during time periods in our analysis when the input is well above that level, such as during the application of critical kicks based on a local minimum of *δ*(*g*). The presence of intrinsic oscillations complicates the case of continuous inputs, because the impact of an input will depend on the phase at which it starts, as in recent phase resetting analysis for bursting models [18,19]. Furthermore, our results about constraints on input times during which spiking can occur in the LIF model with continuous inputs clearly depend on the absence of intrinsic firing.

Unlike the models that we have considered, many other neuronal models have two- or higher-dimensional intrinsic dynamics. Reductions into fast and slow subsystems (e.g., [20,21]) or other reductions [22] offer the possibility of extracting subsystems from these models on which a similar analysis to ours, including the partitioning of an appropriate phase space into bands associated with certain spike numbers in the discrete case, could be performed. It is also possible that results could be obtained after reduction to a firing time map (e.g., [23]). Moreover, analytical techniques can be used to reduce general oscillator models of arbitrary dimension, subject to weak inputs of a prescribed time course, down to forced scalar phase equations [24]. Optimization techniques could be applied to tailor the forcing terms in such equations, within certain constraints, but of course this option is only available if the intrinsic neuronal dynamics is oscillatory and the inputs are weak. Direct simulations can also be done to begin to examine how *δ*(*g*) varies with *g *for particular higher-dimension models. Some preliminary simulations yielded a monotone *δ*(*g*) for a few conductance-based models (e.g. [25-27]) in certain parameter regimes, as well as a non-monotone *δ*(*g*) for a particular scaling of a reduced Hodgkin-Huxley model ([27], Type B^-^), but a thorough exploration of such models remains for future work.

### Neuronal relevance and related issues

Given that we can identify an optimal input structure, it is important to ask whether the similarity of actual input streams to the proposed optimum can be checked experimentally and whether such an optimal input could actually be realized by a network of neurons. As for the former question, membrane potential dynamics can be recorded, including identification of changes in potential associated with synaptic inputs, and associated synaptic input features can be estimated [28], so it appears that the relevant experimental techniques are indeed available.

As for the latter, at least certain features of the optimal inputs we have identified do appear to be achievable. In the discrete input case, consider first a pool of presynaptic neurons providing inputs with the same decay rates, with some distribution of input amplitudes. This pool could be large or small, depending on the brain area involved. The overall input to a postsynaptic cell could be tailored by adjusting the relative firing times of these presynaptic cells. For example, a critical kicks strategy not based on a minimal input threshold could be achieved if a large initial input was given, followed by a sequence of appropriately timed smaller inputs. This input pattern could be realized either through a synchronized input from a group of neurons followed by later inputs from a subset of these neurons or from a distinct group of neurons, perhaps made active by the initial large input as well. Short-term synaptic depression could play a role in this tapering of inputs within the input train. The presence of short-term plasticity would alter the set of input patterns that could be provided, but for a fixed form of postsynaptic dynamics, only certain input patterns would be near-optimal, and if short-term plasticity promoted these, then the loss of the capability to produce non-optimal patterns would be irrelevant. Similarly, neuromodulators also can modulate synaptic dynamics, in a way that differs across different cell types [29-31]. Although we have neglected short-term plasticity and neuromodulation here, considering their effects on optimal input strategies could be an interesting direction for future work. Moreover, if a given presynaptic neuron repeatedly fires in a certain timing relationship with a postsynaptic neuron under an optimal input pattern, then spike timing dependent plasticity (STDP) could also become relevant. Generally, the properties of the inputs received by a given postsynaptic neuron, or the membrane potential variations in that neuron that are induced by inputs, can vary across different situations in real neuronal networks; indeed, these inputs need not come from the same presynaptic source in different states [32]. This observation is consistent with the idea that input patterns could be tailored to achieve different functions.

Given these arguments in favor of the idea that inputs to neurons can indeed be varied in biologically reasonable ways, our work leads to several biological conclusions and predictions. First, the pattern of inputs needed to maximize firing will depend on a neuron's intrinsic dynamics. This point has been discussed in previous theoretical work in the weak or noisy input limit (e.g., [27,33,34]) as well as in some past experimental work [35,36], and we expect this principle to hold quite generally. Second, there may exist an optimal timing following a spike at which the input strength needed to elicit an additional spike is minimized. Again, although we consider excitable neurons receiving strong inputs, this idea is related to past work on PRCs for oscillators receiving weak inputs; this idea differs from the concept of resonance, however, in that the optimal timing would not be solely determined by a postsynaptic neuronal intrinsic frequency. Related to this idea of optimal timing, we might predict that neurons' afterhyperpolarization time courses would differ across brain areas that receive input streams with different characteristics (cf. [37]); that is, the intrinsic dynamics of afterhyperpolarization and network input characteristics could have co-evolved to achieve some form of optimality. Third, we would speculate that perhaps background input levels could be tailored to keep neurons in active brain regions in a regime where *δ*(*g*) is small and hence the barrier to spiking is lowest; attention could even be related to the selection of such a state. Past results have shown that input fluctuations can establish a regime of high spike-time reliability [38,39] or high sensitivity of firing frequency to input current strength [37,40,41], and tuning background input levels to facilitate or suppress postsynaptic activity is at least biologically plausible. Finally, we would also predict that excitatory postsynaptic potential time courses themselves would differ in different neuron types, activity states, developmental stages, and brain areas, as has been seen in experimental work [30,42], in a way that is interrelated with the dynamic properties of the postsynaptic neurons involved.

Admittedly, the precise optimal input patterns that we have found in the discrete case do depend on certain aspects of the postsynaptic neuronal dynamics that might render them hard to achieve. For example, for the LIF model, we found that optimal inputs arise when *v *= *I*, but it is not clear how this information would be available to the input source. Clearly, we are considering a situation where the input source is not directly impacted by the firing of the postsynaptic neuron receiving the inputs; recurrently connected networks are outside the scope of this work. Moreover, stochastic effects could alter the details of any target spike train and synaptic release pattern. It is reasonable to expect that stochastically perturbed versions of the optimal input patterns identified for deterministic models would provide the optimal response distribution in the presence of weak noise, but careful investigation of this issue remains for future work.

## Appendix

Similar methods to those applied to the LIF model with continuous input can be used for the theta model with continuous input. Here we concisely summarize the approach and the main results. Again, we replace *γ*(*t*), given in equation (28), with its time average, and thus we consider the equation

with

on the time interval (*T_a_*, *T_b_*) from one spike to the next. As *θ*(*T_a_*) = -*π *and *θ *(*T_b_*) = *π*,

Combining these equations yields(45)

and spike times *T_i _*and associated values  are found by solving these equations repeatedly.

Lower and upper bounds *t*(*β*),  on firing times are estimated from the solutions of

since *γ*(*t*) > -*b *> 0 is required for spiking to start. Technically, a single final spike can be fired if *γ *rises above -*b *and then falls below it, however, so the upper bound here is not precise.

As with the LIF model, constraints on spike times and changes in spike times with *β *can be explored with these ideas. As one example, consider time *T*_1_(*β*) when the first spike is fired. It can be shown that  as *β *→ ∞ and hence *T*_1_(*β*) → 0 as *β *→ ∞ as well, if it exists. This result yields

but for *T_a _*= *T*_0_, *T_b _*= *T*_1_, the left hand side of equation (45) becomes , a contradiction. Hence, no first spike can occur, and we conclude that spiking in the theta model with input (28) is lost as *β *→ ∞.

## Competing interests

The authors declare that they have no competing interests.
